# Supplementary material for: High level risky sexual behavior among persons living with HIV in the urban setting of the highest HIV prevalent areas in Ethiopia: Implications for interventions
Source: PLoS One. 2020 Nov 25;15(11):e0242701. doi: 10.1371/journal.pone.0242701 (PMC7688102; doi:10.1371/journal.pone.0242701)
Supplement: S1 File — (PDF) [file pone.0242701.s001.pdf]

To the interviewer, please inform the respondent about the aim of the study as described below.

INFORMED CONSENT SHEET

Dear respondent my name is __________ and I am working with researcher from Mettu University who is currently conducting survey on Sexual behavior and associated factors among persons living with HIV in Gambella town, Southwest Ethiopia. As part of this survey, we are collecting information on socio-demographic characteristics; behavioral, social and medical factors; sexual practice and partner related factors. The aim of this study is to investigate sexual practices of clients following their antiretroviral therapy (ART) and factors affecting their behavior. Your genuine response will help to fulfill the knowledge gap about sexual behavior and might be used for further improvement of HIV intervention program by responsible bodies including the health institutions. All information taken from the study will be coded to protect each subject's name. No names or other identifying information will be collected and used when discussing or reporting the data. The investigator(s) will safely keep all files and data collected in a secured locked cabinet in the principal investigators' office. Once the data has been fully analyzed, it will be destroyed. All the information, I am going to collect will be used only for research purposes and kept confidential. The overall questions will not take more than 30 minutes. You have the right not to participate at all or withdraw at the middle of the interview and there is no payment for your participation. Is it clear? (If not clear, repeat the informed consent)

Do you agree to participate?

1. Yes ☐ 2. No ☐

(If yes, continue the interview) (If no respect the decision and thank him/hem)

Mettu University, Faculty of Public health and Medical Sciences

Department of public health

**Mettu University**
**Faculty of Public health and medical sciences**
**Department of public health: Risky sexual behavior questionnaire**
**PI Name:** Fikade Wandimagegn
**Study Title:** High level of risky sexual behavior among persons living with HIV in the urban setting of the highest HIV prevalence in Ethiopia:
**IRB No:** HSFRB/10/19

Participant Code	Data Collector	Date

Read informed consent for risky sexual behavior questionnaire	
Respondent agrees to be interviewed.......................1<br>↓	Respondent does not agree to be interviewed ....2<br>—**end**

<u>**Questionnaire Contents**</u>

Section I: Socio-demographic characteristics of the study participants 3

Section II: Behavioral and Social factors...4

Section III: Sexual practice and Partner related factors... 5

<u>Instructions:</u> For questions which have choices 'tik' (√) responses of respondents in the box provided [ ] and encircle the number in front of the response at the same time. For other questions, write appropriate respondent answer on the space proved in the response columns. In some case, it is not necessary to ask some consecutive question/s based on the participant's response/answer in such cases follow skip pattern provided in the questionnaire.

Section I: Socio-demographic characteristics of the study participants

INTERVIEWER SCRIPT: To begin, we would like to get some basic information about you and your family

Q#	Questions	Responses
101.	How old were you at your last birthday?	_____ _____ Age in completed years
102.	Is the participant a male or a female?<br>*[Answer this question by observation only)*	[ ] Female...................1<br>[ ] Male............2
103.	Have you ever attended any education? [NOTE: any education is include both informal (adult education) and formal education)	[ ] Yes...................1<br>[ ] No............2 → **Skip to 105**
104.	What is the highest level of school you attended: primary, secondary, technical/vocational, or higher?	Education Level<br>[ ] Adult — 1<br>[ ] Primary (grade1-8) — 2<br>[ ] Secondary (grade 9-12) — 3 → **Skip to 106**<br>[ ] Technical/ vocational collage — 4 →**Skip to 106**<br>[ ] Degree & above — 5 → **Skip to 106**
105.	Now I would like you to read this sentence to me.<br><br>SHOW CARD TO RESPONDENT<br>1. The child is reading a book.<br>2. The rains came late this year.<br>3. Parents must care for their children.<br>4. Farming is hard work.<br>IF RESPONDENT CANNOT READ WHOLE SENTENCE, PROBE:<br>Can you read any part of the sentence to me?	[ ] Cannot read at all..........................1<br>[ ] Able to read only parts of sentence..2<br>[ ] Able to read whole sentence……...3<br>[ ] Blind…...5
106.	What is your religion?	[ ] Orthodox............................ 1<br>[ ] Catholic............................... 2<br>[ ] Protestant.............................. 3<br>[ ] Muslim................................. 4<br>[ ] OTHER__________________ 96(SPECIFY)
107.	What is your ethnic group?	[ ] Oromo............................ 1<br>[ ] Anywa............................... 2<br>[ ] Nuer...................................... 3<br>[ ] Amahara................................. 4<br>[ ] OTHER__________________ 96(SPECIFY)
108.	What is your marital status now?<br>[PROBE] Are you currently married or living together with a man as if married?<br>Or, are you single, or are you widowed, divorced, or separated?	[ ] Married or living in union…..1<br>[ ] Single...............2<br>[ ] Widowed...............3<br>[ ] Separated...............4<br>[ ] Divorced...............5
109.	What is your occupational status?	[ ] Governmental employee ………..…1<br>[ ] Student………..………………2<br>[ ] Daily labor …………..….3<br>[ ] Self business ....4<br>[ ] House wife ………..….5<br>[ ] OTHER________________ 96 (SPECIFY)
110.	How many people usually live in this household?	____ ____ People

	[NOTE: *If a person stays half of the week in the household and shares food from same pot then s/he should be considered as household member]	
111.	How much your monthly average income in Ethiopian birr?	**_____________(in ETB)**

Section II: Behavioral and Social factors

INTERVIEWER SCRIPT: Now I would like to talk about behavioral and social factors – Alcohol use, cigratte use, khat use, desire of child, HIV prevention and stigma

Q#	Question		Response	
201.	Did you have any alcoholic beverage consumption in the last one month?		[ ] yes …………1<br>[ ] no ………….2→ **Skip to Q# 203**	
202.	Number of drinks	Gender	Average daily consumption	Average weekly consumption
		Female	[ ] ≤ 3 drinks in a day………...1<br>[ ] > 3 drinks in a day ……….2	[ ] ≤ 7 drinks in a week……….1<br>[ ] > 7 drinks in a week……….2
		Male	[ ] ≤ 4 drinks in a day…...........1<br>[ ] > 4 drinks in a day ……….2	[ ] ≤ 14 drinks in a week……...1<br>[ ] > 14 drinks in a week……...2
203.	Did you smoke cigarette in the last one month?		[ ] yes …………1<br>[ ] no ………….2 → **Skip to Q# 205**	
204.	Did you smoke at least one cigarette per day/at least seven cigarette per a week?		[ ] yes …………1<br>[ ] no ………….2	
205.	Did you chew 'khat' in the last one month?		[ ] yes …………1<br>[ ] no ………….2	
206.	Did you used other kind substance in the last one month?<br>(Eg. Shisha, hashish (marijuana), cocaine, benzene etc.)		[ ] yes …………1___________________________________(specify)<br>[ ] no ………….2	
207.	Do you have a child?		[ ] yes………..1<br>[ ] no………..2 → **Skip to Q# 209**	
208.	Is there any HIV positive child/children in your family?		[ ] yes………..1<br>[ ] no………..2	
209.	Do you have a desire for more child/children in the future?		[ ] yes………..1<br>[ ] no………..2 → **Skip to Q# 211**	
210.	How many children you desired?		___________ (specify in number)	

211.	Have you been pregnant in the past 12 months<br>(Note: ask only females participants)	[ ] yes …………1<br>[ ] no …………2→ **Skip to Q# 213**
212.	Was the pregnancy is intended?	[ ] yes …………1<br>[ ] no …………2
213.	Have you caused a pregnancy in the past 12 months?<br>(Note: ask only males participants)	[ ] yes …………1<br>[ ] no …………2 → **Skip to Q# 215**
214.	Was the caused pregnancy is intended?	[ ] Yes…………1<br>[ ] No…………..2
215.	Did you attended any support group discussion on HIV prevention?	[ ] Yes…………1<br>[ ] No…………..2
216.	Did you received any skills' building training on safer sex practice such as (condom use, negotiation skills …)	[ ] Yes…………1<br>[ ] No…………..2
217.	Did you received any education and/or counseling on protecting self from another HIV strain	[ ] Yes…………1<br>[ ] No…………..2
218.	Had you experienced perceive stigma?<br>**[PROBE]** felt self-blame, shame, lack of disclosure, reduced self-confidence, loss of motivation, withdrawal from social contact and health-based interactions, abandonment of planning for the future …etc	[ ] yes …………1<br>[ ] no ………....2
219.	Had you experienced enacted stigma?<br>**[PROBE]** excluded from a social gathering, abandoned by your spouse/partner, isolated in your household, no longer visited or visited less frequently by family and friends, teased and/insulted, lost customers to buy produce/goods or lost a job, lost housing or not been able to rent housing, denied religious rites/services, threatened with violence …etc	[ ] yes …………1<br>[ ] no ………....2

Section III: Sexual practice and Partner related factors

INTERVIEWER SCRIPT: Now I would like to talk about sexual practice and partner related issues-sexual partner, type of partner, partner HIV status and condom use

Q#	Questions	Responses			
301.	Had sexual partner in the past three months?	[ ] yes …………1<br>[ ] No …………2 → **skip Q# 311**			
302.	Number of sexual partner/s in the past three months	[ ] ___ ___(in number)			
303.	What type of sexual partner/s you have in the past three months?	[ ] Steady …………1<br>[ ] Causal …………2<br>[ ] Both …………..3			
304.	HIV status of your partner	Partner 1	Partner 2	Partner 3	Partner 4

	Note: use partner 2-4 columns in case multiple partner	[ ] Negative …1<br>[ ] Positive …. 2<br>[ ] Unknown …3	[ ] Negative …1<br>[ ] Positive …. 2<br>[ ] Unknown …3	[ ] Negative …1<br>[ ] Positive …. 2<br>[ ] Unknown …3	[ ] Negative …1<br>[ ] Positive …. 2<br>[ ] Unknown …3

305.	Did you discuss about safe sex with your partner/s?	[ ] yes ....1<br>[ ] no ……………2
306.	How many months you stay with current partner?<br>(**Note:** in case of multiple partner at data collection date fill the longest stay)	___ ___ (in months)
307.	Did you disclose your HIV status to your partner?	[ ] yes……………1<br>[ ] no…………….2
308.	Do you have condom/s at your home	[ ] yes …………1<br>[ ] no ………….2
309.	What kind of condom use pattern in the past 3 months?<br>(**Note:** always mean using condoms during every sex act in the last 3 months)	[ ] always……………1→ **skip Q# 311**<br>[ ] sometimes ………..2<br>[ ] Not at all ……...... 3
310.	What is the main reason to use condom sometimes or not use at all?	[ ] partner refusal…………………………..….1<br>[ ] partner already had HIV………………..…..2<br>[ ] condom decrease pleasure…………………...3<br>[ ] condom an available…………………………4<br>[ ] was drank and didn't use……………………5<br>[ ] wanted to have child……………………..…6<br>[ ] lack of knowledge…………………………..7<br>[ ] I am already infected…...…………………..8<br>[ ] Other……………………………………96 (specify)
311.	Length of follow up at ART clinic<br>(**Note:** Use client treatment identification number to fill from registered document)	___ ___ (in moths)
312.	Current CD4 count<br>(**Note:** Use client treatment identification number to fill from registered document)	[ ] $\leq$ 350 cells/mm^3 ……………..2<br>[ ] < 350 cells/mm^3 ………………2
