# Supplementary material for: High level risky sexual behavior among persons living with HIV in the urban setting of the highest HIV prevalent areas in Ethiopia: Implications for interventions
Source: PLoS One. 2020 Nov 25;15(11):e0242701. doi: 10.1371/journal.pone.0242701 (PMC7688102; doi:10.1371/journal.pone.0242701)
Supplement: S2 File — (PDF) [file pone.0242701.s002.pdf]

ለመረጃ ሰብሳቢዎች፡- እባካችሁ ለጥናቱ ተሳታፊዎች የጥናቱን አላማና ጭብጥ ከታች በተገለፀው መልኩ አስረዷቸው።

በመረጃ ላይ የተደገፈ የስምምነት ወረቀት

ውድ ተሳታፊ ሥሜ……………………………………………… ይባላል። እኔ የምሰራውም በደቡብምስራቅ ኢትዮጵያ በጋምቤላ ከተማ ኤችአይቪ በደማቸው ያለባቸው የማህበረሰበ ክፍሎች ለሚያደርጉት ጥንቃቄ የጎደለው(ጾትዊ) የግብረስጋ ግንኙነት ለኤችአይቪ መዛመት ያለው አስተዋፅኦና ተያያዥ ችግሮቹን በተመለከተ ጥናትና ምርምር ከሚሰራው ከመቱ ዩኒቨረስቲ ጋር ነው። በዚህ ጥናት ክፍል ውስጥ የምናካትታቸው /የምንሰበስባቸው መረጃዎች የአካባቢያዊና ባህላዊባህሪያት፡ ህክምና እና ስነ ባህሪዊ እናማህበራዊ(ባህላዊ) እክሎች(ተጽዕኖዎች): ከግብረ ስጋ ግንኙነትና ከትዳር አጋር ጋር የተገናኙእክሎች(ተጽዕኖዎች) ናአው። የዚህ ጥናት አላማ የኤችአይቪ ቫይረስ ህክምና የሚከታተሉ ተሳታፊዎችን የፆታዊ ግንኙነት ልምድ መገምገምና በባህሪያቸው ላይ ተፅዕኖ ሊያመጡ የሚችሉ ምክንያቶችን መረዳት ነው። የእርሶ ትክክለኛ ምላሽ ከግብረስጋ ግንኙነት ስነባህሪያት ጋር በተያያዙ ያሉ የዕውቀት ክፍተቶችን ከመሙላት ባለፈ በኤችአይቪ መከላከል ላይ ለሚሰሩ አካላት ጤና ተቆማትን ጨምሮ ማሻሻያዎችን እንዲያደርጉ ያግዛል። ከተሳታፊዎች የሚገኙት ሁሉም ማስረጃዎች በኮድ እንዲቀመጡ ይደረጋል። ይህም የተሳታፊዎችን ስም ላለመጠቀም ይረዳል። በዉይይትም ሆነ በሪፖርት ወቅት የተሳታፊ ስም አይገልጽም።.የመረጃ ሰብሳቢው የሰበሰበውን ህሉንም መረጃ በዋና መረጃ ስብሳቢው ቢሮ በጥንቃቄ በተቆላፊ ሳጥን ውስጥ ቆልፎ ያስቀምጣል። መረጃዎቹ አንድ ጊዜ ተመርምረው/ተጠንተው ከተጠናቀቁ በኍላ በጥንቃቄ ይወገዳሉ ።እኔ የምሰበስበው መረጃ ሁሉ ለጥናታዊ ምርምር ብቻ የሚውል እና በሚስጥር የሚጠበቅ ነው ።አጠቃላይጥያቄው ከ 30 ደቂቃ በላይ አይወስድም ለዚህ ቃለ መጠይቅ በመሳተፍሀወ/ሽ ክፍያ የለውም። በፈለገህ/ሽ ጊዜ ማቋረጥ ወይም ሙሉ ለሙሉ አለማሳትፍ መብቱ ያንተ/ቺ ነው። ግልፅ ነው? (ግልፅ ካልሆነ በመረጃላይ የተደገፈ ስምምነቱን ድገም/ሚ)

ለመሳተፍ ተስማምተሀል/ሻል? 1. አዎ [ ] 2. አልተስማማሁም [ ]

የተሳታፊው/ዋ መልስ አልተስማማሁም ከሆነ ውሳኔውን/ዋን አክብረህ/ሽ አመስግነህ/ሽ ሸኝ/ኚ። ምላሹ/ሿ አዎ ከሆነ ቃለመጠይቁን ቀጥል/ይ።

በመቱ ዩኒቨርሲቲ በህብረተሰብ ጤና እና በህክምና ፋካሊቲ
የህብረተሰብ ጤና ክፍል

በመቱ ዩኒቨርሲቲ በህብረተሰብ ጤና እና በህክምና ፋካሊቲ
በህብረተሰብ ጤና ክፍል: ጥንቃቄ ስለጎደለው(የተጋለጠ) ፆታዊ ግንኙነት የተዘጋጀ መጠይቅ
የዋና ተመራማሪ ስም: ፋቃደ ወንድምአገኝ ዘበነ
የጥናቱ ርእስ፡ በኢትዮጵያ ውስጥ ከፍተኛ ስርጭት ካለባቸው ከተሞች አኳያ ከኤችአይቪ ጋር ከሚኖሩ ሰዎች በከፍተኛ ደረጃ የተጋለጠ የግብረስጋ ግንኙነት ባህሪ
ጥናቱ የፀደቀበት ቁጥር፡ HSFRB/10/19

የተሳታፊዎች መለያ	**መረጃ** ሰብሳቢዎች	ቀን

ጥንቃቄ ስለጎደለው ፆታዊ ግንኙነት የተዘጋጀውን መጠይቅ እንዲሞሉ የተጠየቁ ግለሰቦች<br><br>**የተጋለጠ የግብረስጋ ግንኙነት ባህሪ ጥያቄ የመግባብያ ሰነዱን አንብብ**	
ማላሽ ለመስጠት የተስማሙ 1	ምላሽ ለመስጠት ያልተስማሙ ....2 ── **አቁም**

የመጠይቁ ማውጫ

ክፍል I ፡ የጥናቱ ተሳታፊዎች አካባቢያዊና ባህላዊባህሪያት...3

ክፍል II:ስነ ባህሪዊና ማህበራዊ(ባህላዊ) እክሎች(ተጽዕኖዎች)..4

ክፍል III: ከግብረ ስጋ ግንኙነትና ከትዳር አጋር ጋር የተገናኙእክሎች(ተጽዕኖዎች) 5

<u>መመሪያ:</u> ከታች ለተዘረዘሩት መጠይቆች የራይት (✔) ምልክት በተዘጋጀው ሳጥን ወይም [ ] ምልክት ውስጥ በማስቀመጥና በተመሳሳይ በፊት ለፊቱ የተሰጠውን ቁጥር በማክበብ መምረጥ ይቻላል። ለተወሰኑት መጠይቆች ደግሞ ተገቢውን ምላሽ በተሰጠው ቦታ ላይ በመፃፍ መግለፅ ይቻላል። በአንዳንድ ምክንያቶች በተሳታፊው ምላሽ ላይ በመመርኮዝ ተያያዥ ጥያቄዎችን ላለማቅረብ ጥረት ከመደረጉም በተጨማሪ እንደ ተሳታፊዎቹ ምላሽ አይነት ቀጣዩን ጥያቄ መመለስ ሳያስፈልጋቸው እንዲያልፉት ተደርጓል።

ክፍል 1: የጥናቱ ተሳታፊዎች አካባቢያዊና ባህላዊ ባህሪያት

የጠያቂው መግለጫ: ለመጀመር ያክል ስለራስህ/ሽ እና ስለ ቤተሰቦችህ/ሽ ሁኔታ መጠነኛ መረጃ ብትሰጠኝ/ጪኝ።

ጥ.ቁ	ጥያቄዎች	ምላሾች
101.	የመጨረሻ ልደትህን/ሽን ስታከብር/ሪ ስንት አመትህ/ሽ ነበር?	____ ____ እድሜ በተጠናቀቀው አመት
102.	*የተሳታፊያችን ፆታ ምንድን ነው?*<br>*[ይህ ጥያቄ በምልከታ ብቻ ይመለስ]*	[ ] ሴት...................1<br>[ ] መንድ............2
103.	ትምህርት ተምረሀል/ሻል?<br>[መግለጫ: ትምህርት ሲባል መደበኛ ወይም ኢ-መደበኛ (መሰረተ ትምህርት) ሊሆን ይችላል።	[ ] አዎ....................1<br>[ ] የለም.............2 → **ወደ ጥያቄ 105 እለፍ/ፊ**
104.	ከፍተኛ የትምህርት ደረጃህ/ሽ ምንድን ነው?<br>[መጀመሪያ ደረጃ፣ ሁለተኛ ደረጃ፣ ቴክኒክና ሞያ፣ ድግሪ	የትምህርት ደረጃ<br>[ ] የጎልማሶች ትምህርት — 1<br>[ ] መጀመሪያ ደረጃ (ከ1-8 ክፍል) — 2<br>[ ]ሁለተኛ ደረጃ (9-12 ክፍል) — 3→ **ወደ ጥያቄ 106 እለፍ/ፊ**<br>[ ] ቴክኒክና ሞያ — 4 →**ወደ ጥያቄ 106 እለፍ/ፊ**<br>[ ] ድግሪና ከዛ በላይ — 5 → **ወደ ጥያቄ 106 እለፍ/ፊ**
105.	.አሁን እነዚህን ዓ.ነገሮች እንድታነብልኝ/ቢልኝ እፈልጋለሁ። ለተሳታፊው/ዋ ካርድ የተፃፉትን ዓ.ነገሮች አሳይ<br>1. ልጁ/ልጇቷ መፅሀፍ እያነበበች ነው።<br>2. በዚህ ዓመት ዝናቡ ዘግይቶ ነው የመጣው።<br>3. ወላጆች ለልጆቻቸው ጥንቃቄ ማድረግ አለባቸው።<br>4. ግብርና ከባድ ስራ ነው።<br>ተሳታፊዎች ሁሉንም ዓ.ነገሮች ማንበብ ካልቻሉ፣<br>**ምርመራ**: ከዓ.ነገሮቹ ልታነበው የምትችለው ቃል ይኖራል?	[ ] ሁሉንም ማንበብ አልችልም................................1<br>[ ] የተወሰኑ ቃላቶችን አነባለሁ.......................................2<br>[ ] ሁሉንም ዓ.ገሮች ማንበብ እችላለሁ……...3<br>[ ] ማየት አልቻልኩም…….................................5
106.	የምን ሀይማኖት ተከታይ ነህ/ሽ	[ ] ኦርቶዶክስ............................ 1<br>[ ] ካቶሊክ.............................. 2<br>[ ] ፕሮቴስታንት............................. 3<br>[ ] ሙስሊም................................ 4<br>[ ] ሌላ________________ 96(ይገለፅ)
107.	የየትኛው ጎሳ አባል ነህ?	[ ] ኦሮሞ............................ 1<br>[ ] አኙዋክ.............................. 2<br>[ ] ኑዌር..................................... 3<br>[ ] አማራ................................ 4<br>[ ] ሌላ________________ 96(ይገለፅ)
108.	በአሁኑ ወቅት የጋብቻ ሁኔታህ/ሽ ምን ይመስላል? አግብተሀል/ሻል፣ እንደ ትዳር በማሰብ ከጓደኛህ/ሽ ጋር አብረህ/ሽ እየኖርክ/ሽ ነው፣ አላገባህም/ሽም፣ ሚስትህ/ባልሽ ሞታለች/ሞቷል፣ ተፋተሀል/ሻል ወይስ ተለያይተሀል/ሻል?	[ ] አግብቻለሁ /አብረን እየኖርን ነው…..1<br>[ ] አላገባሁም................2<br>[ ] ባለቤቴ ሞታለች/ሞቷል................3<br>[ ] S ተለያይተናል...............4<br>[ ] ተፋተናል...............5
109.	የስራ ሁኔታ	[ ] የመንግስት ተቀጣሪ…..….1<br>[ ] ተማሪ……..……………….2<br>[ ] የቀን ሰራተኛ…………..….3<br>[ ] የግል ስራ...............4

		[ ] የቤት እመቤት……..….5<br>[ ] ሌላ________________ 96 (ይገለፅ)
110.	በዚህ ቤተሰብ ውስጥ ምን ያህል ሰው ይኖራል?<br>**መግለጫ ፡** በቤተሰቡ ውስጥ ለግማሽ ሳምንት ያክል ምግብ እና መሰል ነገሮችን በጋራ እየተጠቀመ የቆየ ሁሉ እንደ ቤተሰቡ አባል ይቆጠራል።	____ ____ ሰዎች
111.	አማካይ ወርሀዊ ገቢህ/ሽ በኢትዮጵያ ብር ስንት ነው?	______________(በኢትዮጵያ ብር)

ክፍል 2 ፡ስነ ባህሪያዊ እና ባህላዊ እክሎች(ተፅዕኖዎች)

አሁን ስለ ስነ-ባህሪያዊ እና ማህበራዊ(አካባቢያዊ) ተፅዕኖዎች እናወራለን። አልኮል ስለመጠቀም፣ ሲጋራና ጫት ስለመጠቀም፣ ልጅ ሰለመውለድ ፍላጎት፣ ኤችአይቪን ስለመከላከል እና ስለ መገለል

ተ.ቁ.	ጥያቄ		መልስ	
201.	በባለፈው ወር ውስጥ አልኮል ተጠቅመህ/ሽ ተውቃለህ/ሽ		[ ] አዎ…………1<br>[ ] አልተጠቀምኩም ………….2→ **ወደ ጥያቄ 203 እለፍ/ፊ**	
202.	የመጠጥ ብዛት	ፆታ	አማካይ የቀን ፍጆታ	ሳምንታዊ አማካይ ፍጆታ
		ሴት	[ ] 3 ወይም ከዛ ያነሰ…….1<br>[ ] በቀንከ 3 በላይ 2	[ ] በሳምንት 7 ወይም ከዛ በታች….1<br>[ ] ከ7 በላይ ……….2
		Male<br>ወንድ	[ ] 4 ወይም ከዛ በታች…...........1<br>[ ] ከ 4 በላይ ……….2	[ ] በሳምንት 14 ወይም ከዛበታች……1<br>[ ] ከ14 በለላይ ……...2
203.	ባለፈው አንድ ወር ውስጥ ሲጋራ አጭሰህ/ሽ ታውቃለህ/ሽ		[ ] አዎ…………1<br>[ ] አላጨስኩም………….2 → **205 ወደ ጥያቄ 205 እለፍ**	
204.	በየቀኑ ቢያንስ አንድ ሲጋራ ታጨስ ነበር?		[ ] አዎ…………1<br>[ ] አላጨስም………….2	
205.	ባለፈው አንድ ወር ውስጥ ጫት ቅመህ/ሽ ታውቃለህ/ሽ		[ ] አዎ…………1<br>[ ] አልቃምኩም………….2	
206.	አንድ ወር ውስጥ ሌሎች እፆችን ተጠቅመህ/ሽ ታውቃለህ/ሽ?<br>**ለምሳሌ**: ሺሻ፣ ሀሺሽ(ማሪዋና)፣ ኮኬን፣ ቤንዚን የመሳሰሉትን		[ ]አዎ…………1_________________________________(ይገለፅ)<br>[ ] አልተጠቀምኩም………….2	
207.	ልጆች አሉህ/ሽ?		[ ] አዎ………..1<br>[ ] የለኝም………..2 → **ወደ ጥያቄ 209 እለፍ/ፊ**	
208.	በቤተሰብህ/ሽ ውስጥ ከኤችአይቪ ጋር የሚኖር ልጅ ወይም ልጆች አሉህ/ሽ?		[ ] አዎ………..1<br>[ ] የለኝም………..2	

209.	ወደፊት ተጨማሪ ልጅ ወይም ልጆች እንዲኖሩህ/ሽ ትፈልጋለህ/ጊያለሽ?	[ ] አዎ………..1<br>[ ] አልፈልግም………..2 → **ወደ ጥያቄ 211 እለፍ/ፊ**
210.	ምን ያህል ልጆች እንዲኖሩህ/ሽ ትፈልጋለህ/ጊያለሽ?	____________ (በቁጥር ይገለፅ)
211.	ባለፉት 12 ወራት ውስጥ እርግዝና ተከስቶብሽ ነበር? (መግለጫ: ለሴት ተሳታፊዎች ብቻ)	[ ] አዎ…………1<br>[ ] አልተከሰተም …….2→ **ወደ ጥያቄ 213 እለፍ/ፊ**
212.	እርግዝናው የታቀደ ነበር?	[ ] አዎ…………1<br>[ ] አልነበረም ………….2
213.	ባለፉት 12 ወራት ወስጥ አስረግዘህ ታውቃለህ? (መግለጫ: ለወንድ ተሳታፊዎች ብቻ የሚጠየቅ።)	[ ] አዎ…………1<br>[ ] አላስረገዝኩም…….2 → **ወደ ጥያቄ 215 እለፍ/ፊ**
214.	እርግዝናው እንዲፈጠር ቀድመህ አቅደህ ነበር?	[ ] አዎ………….1<br>[ ] አላቀድኩም…………..2
215.	ኤችአይቪን በመከላከል ላይ ባተኮሩ ውይይቶች ተሳትፈህ/ሽ ታውቃለህ/ሽ?	[ ] አዎ………….1<br>[ ] አላውቅም…………..2
216.	ጥንቃቄ የተሞላበት ግብረስጋ ግንኙነትን በተመለከተ የአቅም ግንባታ ስልጠና ወስደህ/ሽ ታውቃለህ/ሽ? (ኮንዶም አጠቃቀም፣የመደራደር አቅም...)	[ ] አዎ………….1<br>[ ] አልወሰድኩም…………..2
217.	ኤችአይቪ ሊተላለፍባቸው ከሚችሉ መንገዶች እንዴት መከላከል እንደምትችል/ችይ ትምህርት ወይም የምክር አገልግሎት ወስደህ/ሽ ታውቃለህ/ሽ?	[ ] አዎ………….1<br>[ ] አልወሰድኩም…………..2
218.	አእምሮአዊ የመገለል ችግር አጋጥሞህ/ሽ ያውቃል?<br>[**ምርመራ**] ራስን መውቀስ፣ሀፍረት፣ራስን መግለፅ አለመቻል፣በራስ መተማመን መቀነስ፣ ተነሳሽነት ማጣት፣ከማህበራዊ እና ጤና ማዕቀፎች መገለል፣ ለወደፊት ከማቀድ ራስን መግታት የመሰሰሉት።	[ ] አዎ…………1<br>[ ] አላጋጠሙኝም………....2
219.	አካላዊ የመገለል ችግር አጋጥሞህ/ሽ ያወቃል?<br>[**ምርመራ**] ከማህበራዊ ግንኙነቶች መገለል፣ በትዳር አጋር/ፍቅረኛ መተው፣ በቤት ውስጥ የመለየት፣ በቤተሰብና ጓደኞችበበቂ ሁኔታ ያለመጎብኘት ወይም ጭራሽ አለመጎብኘት፣ ስድብና መንጓጠጥ፣ ስራ ማጣት፣ ደንበኛ መቀነስ ወይም ጭራሽ መጥፋት፣ የኪራይ ቤት መከልከል፣ የአምልኮ አገልግሎት ማጣት፣ የመሰሰሉት	[ ] አዎ…………1<br>[ ] አላጋጠመኝም………....2

ክፍል 3: የግብረ ስጋ ግንኙነት ልማድ እና ከትዳር አጋር ጋር የተገናኙ እክሎች

የጠያቂው መግለጫ: አሁን ስለ ግብረ ስጋ ግንኙነት ልማድና የግብረ ስጋ ግንኙነት አጋር ፆታዊ አጋር፣ የአጋር አይነቶች፣ የፆታዊ አጋርየኤችአይቪ ሁኔታ እና ኮንዶም አጠቃቀምን በተመለከተ እናወራለን።

ጥ.ቁ	ጥያቄዎች	ምላሾች
301.	ባለፉት ሦሥት ወራት ፆታዊ አጋር ኖሮህ/ሽ ያውቃል?	[ ] አዎ………….1<br>[ ] አልነበረኝም…….2 → **ወደ ጥያቄ 311 እለፍ/ፊ**
302.	ባለፉት ሦሥት ወራት ምን ያህል ፆታዊ አጋሮች ነበሩህ/ሽ?	[ ] ___ ___(በቁጥር ይገለፅ)
303.	ባለፉት ሦሥት ወራት ምን አይነት ፆታዊ አጋር/ሮች ነበረህ/ሽ (ነበሩህ/ሽ)	[ ] ተደጋጋሚ …………1

		[ ] ምክንያታዊ…………2<br>[ ] ሁለቱም …………..3			
304.	የፆታዊ አጋርህ/ሽ ኤችአይቪ ሀኔታ ምን ይመስላል?<br>መግለጫ: ምናልባት ከ1 በላይ አጋር ካለህ/ሽ ከ2-4 ያለውን አምድ ተጠቀም/ሚ።	አጋር 1	አጋር 2	Partner አጋር 3	Partner አጋር 4
		[ ]የለበትም/ባትም..1<br>[ ] አለበት/ባት…2<br>[ ] ተመርምሮ/ራ አላወቀም/ችም……3	[ ]የለበትም/ባትም..1<br>[ ] አለበት/ባት…2<br>[ ] ተመርምሮ/ራ አላወቀም/ችም….3	[ ]የለበትም/ባትም1<br>[ ] አለበት/ባት…2<br>[ ] ተመርምሮ/ራ አላወቀም/ችም…3	[ ] የለበትም/ባትም….1<br>[ ] አለበት/ባት…..2<br>[ ]ተመርምሮ/ራ አላወቀም/ችም…..3
305.	ከፆታዊ አጋርህ/ሽ ጋር ጥንቃቄ ስለሞላበት ግብረ ስጋ ግንኙነት ተወያይታችሁ ታውቃላችሁ?	[ ] አዎ................1<br>[ ] አናውቅም……………2			
306.	አሁን ካላችው/ካለው ፆታዊ አጋርህ/ሽ ጋር ለምን ያህል ወራት አብራችሁ ቆያችሁ?<br>(**<u>መግለጫ:</u>** ምናልባት መረጃዉ በምሰበሰብበት ቀን ብዙ አጋሮች ካሉህ/ሽ ረጅም ጊዜ አብራህ/ሮሽ የቆየችውን/የቆየውን ሙላ/ዪ)	___ ___ (በወራት ይገለፅ)			
307.	ለፆታዊ አጋርህ/ሽ ስለራስህ/ሽ HIV ሁኔታ አሳውቀሀል/ሻል?	[ ] አዎ……………1<br>[ ] አላሳወኩም…………….2			
308.	በቤትህ/ሽ ኮንዶም አለህ/ሽ?	[ ] አዎ …………1<br>[ ] የለኝም………….2			
309.	ባለፉት ሦሥት ወራት ለምን ያህል ጊዜ ኮንዶም ተጠቅመሀል/ሻል?<br>**<u>መግለጫ:</u>** ሁልጊዜ ማለት በማንኛውም ፆታዊ ግንኙነት ጊዜ ኮንዶም ተጠቅሜያለሁ።	[ ] ሁልጊዜ……………1→ **ወደ ጥያቄ 311 እለፍ/ፊ**<br>[ ] አልፎ አልፎ………..2<br>[ ] ተጠቅሜ አላውቅም……...... 3			
310.	ኮንዶምን አልፎ አልፎ የምትጠቀምበት/ሚበት ወይም ጭራሽ የማትጠቀምበት/ሚበት ምክንያት ምንድን ነው?	[ ] የፆታዊ አጋሬ ፈቃደኛ አለመሆን………………………….…1<br>[ ] አጋሬም ኤችአይቪ ስላለባት/በት……………….…..2<br>[ ] ኮንዶም እርካታን ስለሚቀንስ…………...3<br>[ ] ኮንዶም ስለማላገኝ…………………………4<br>[ ] ጠጥቼ ስለሚሆንና ስለማልጠቀም…………5<br>[ ] ልጅ እንዲኖረኝ ስለምፈልግ……………………..…6<br>[ ] በዕውቀት ማነስ…………………………..7<br>[ ] ቀድሞም ኤችአይቪ ስላለብኝ…..…………………8<br>[ ] ሌላ………………………………………96 (ይገለፅ)			
311.	በART ክሊኒክ ለምን ያህል ጊዜ ክትትል አድርገሀል/ሻል?<br>**<u>መግለጫ:</u>** የደንበኛ እንክብካቤ መለያ ቁጥርን በመጠቀም ከመዝገብ ላይ ይሞላ	___ ___ (በወራት ይገለፅ)			
312.	ወቅታዊ የCD4 መጠን<br>**<u>መግለጫ:</u>** የደንበኛ እንክብካቤ መለያ ቁጥርን በመጠቀም ከመዝገብ ላይ ይሞላ	[ ] $\leq$ 350 cells/mm^3 …………….2<br>[ ] < 350 cells/mm^3 ………………2			
