# Supplementary material for: High level risky sexual behavior among persons living with HIV in the urban setting of the highest HIV prevalent areas in Ethiopia: Implications for interventions
Source: PLoS One. 2020 Nov 25;15(11):e0242701. doi: 10.1371/journal.pone.0242701 (PMC7688102; doi:10.1371/journal.pone.0242701)

Dear Editor:

I appreciate the comments and suggestions of the academic editor and reviewers; their comments and suggestions were constructive and have improved our manuscript substantially. Accordingly, I have incorporated almost all of the comments and suggestions given by the academic editor and reviewers in the manuscript. Furthermore, I have responded to each of the points raised by the reviewers as follows:

Sincerely,

Tsegaye Berkessa (Corresponding author)

Academic Editor:

- The manuscript still has many typos and grammatical errors. Please make sure that it is thoroughly edited by someone fluent in English. Please also correct the errors listed below

  Response: The comment was accepted and the manuscript was edited.

- Please acknowledge that the sample size calculation was only made for estimating prevalence of risky sexual practice and not for estimating the factors associated with the outcome of interest. This should be discussed as a limitation under the discussion section.

  Response: This comment has been well taken and discussed in the limitation part.

- Line 137: It is not clear how the variable "condom use practice" is considered both as dependent and intendent variable.

  Response: The comment was accepted and all variables that measures outcome variable were removed from the list.

- The data analysis section is superficial and does not tell how the independent variables were screened for the multivariable model.

  Response: The comment was accepted and revision was made accordingly. We used model-building steps mentioned by Hosmer and Lemeshow in applied

logistic regression book. All the variables with p-value < 0.25 with risky sexual practice in unavailable analysis were fitted to the final multivariable logistic regression model.

Abstract

- Line 21: Reconsider the use of the word "edge"

  Response: The comment was accepted and we replaced by another word.

- Line 26: "characters" >> "practices"

  Response: The comment was accepted and correction was made accordingly

- Line 28: "multivariate" >> "multivariable" (please do the same throughout the document)

  Response: The comment was accepted and revision throughout the manuscript was made accordingly

- Line 35: "were" >> "have"

  Response: The comment was accepted and correction was made accordingly

- Line 44 reconsider the use of the word "eradication"

  Response: The comment was accepted and correction was made accordingly.

Background

- Line 49 "were" >> "had"

  Response: This comment has been well taken and corrected accordingly.

- Line 60-62: The sentence is not clear

  Response: The comment was accepted and the sentence was revised.

- Line 74-75: what do you mean by "sexual exchange"? its not clear.

  Response: The comment was accepted and the phrase was clarified.

- Line 85-87: please rephrase the sentence.

  Response: The comment was accepted and the sentence was revised.

- Line 91: Reconsider the use of the word "edge"

  Response: The comment was accepted and we replaced by another word.

Methods

- Line 96: "18 years and above" >> "18 years or above"

Response: Response: Correction was made accordingly

- Line 98-100: Please remove the following sentence "The town is bordered by Gambella district and Bonga village by East, Oromia Regional State by Northeast, Gambella district by Northwest and West, and Abobo district by South."

  Response: The comment was accepted and the sentence was removed from the manuscript.

- Line 109-111: remove the header "study population" and put the sentence "The study populations were all HIV positive adults attending ART from June to July 2019 at ART clinics in Gambella town." as the first sentence of the "sample size and sampling procedure" header.

  Response: The comment was accepted and revised accordingly.

- Line 115: please remove "CI". Please note that confidence level and confidence interval are different.

  Response: This is definitely correct and we have modified accordingly.

- Line 126 : "if" >> "when"

  Correction was made accordingly

- Line 127: Please mention the local language

  Response: The comment was accepted and local language was mentioned.

- Line 130: "characters" >> "practices"

  Response: Revision was done accordingly

Results

- Table 1: please arrange all categories of the variables in decreasing order with the exception of those variables that have natural order (monthly income, family size)

  Response: The comment was accepted and revised accordingly.

- Line 174: please reconsider the use of the word "were"

  Response: This comment has been well taken and corrected accordingly.

- Table 2: what do you mean by "Caused intended", please also indicate sample size for the variables.

  Response: "Caused intended" mean males who caused the pregnancy and reported that they intended to cause the pregnancy. This comment was accepted

Tsegaye Berkessa Tola

and this variable was removed from the manuscript due to sample size insufficiency, less than 10 events per variable (EPV).

- Table 3: Bivariate >> Bivariable, Multivariate >> multivariable (please also do the same throughout the document)

  Response: The comment was accepted and revised accordingly.

- Table 3: it is not clear how the candidate variables were identified for the multivariable model, This must be described in the data analysis sub-section

  Response: The comment was accepted and revised accordingly.

- Table 3: Shisha/hashish use3x??? what do you mean by 3X?

  Response: It is editorial error. The comment was accepted and revised accordingly.

Discussion

- Line 275-6: The sentence is not clear.

  Response: The comment was accepted and the sentence was revised.

- Line 284-85: Please remove the sentence "First, alcohol consumption in this study was not verified by using Alcohol Use Disorder Identification Test (AUDIT).". It is not a must to use this tool

  Response: The comment was accepted and the sentence was removed from the document.

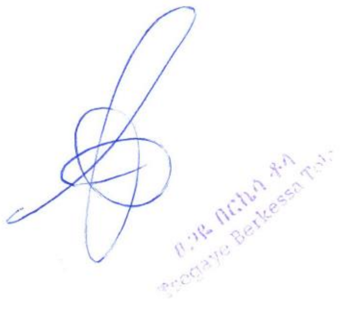

Supplement: Attachment — Submitted filename: Response to Reviewers.pdf [file pone.0242701.s006.pdf]
