# Supplementary material for: High level risky sexual behavior among persons living with HIV in the urban setting of the highest HIV prevalent areas in Ethiopia: Implications for interventions
Source: PLoS One. 2020 Nov 25;15(11):e0242701. doi: 10.1371/journal.pone.0242701 (PMC7688102; doi:10.1371/journal.pone.0242701)
Supplement: Attachment — Submitted filename: Review feedback.docx [file pone.0242701.s003.docx]

General feedback:

- The scientific writing and English language needs editorial work.
- There is major flaw in the operational definition of respondents’ sexual behavior/practice which significantly affects the study finding, discussion and conclusion sections alike.

Specific feedbacks:

- The HIV prevalence in Ethiopia is technically “concentrated” (less than 1%) according to UNAIDS definition while the average in Africa is “generalized” 5% as indicated in the background of the manuscript (Line 50). Nonetheless, the authors characterized Ethiopia’s HIV epidemic as one of the highest in Africa which is not correct (see the Abstract and line 51 without any reference).
- This study has critical deficiency/limitation on the operational definition of “risky sexual behavior/practice” (specifically the consistent condom use …line 116) which I believe leads to remarkable challenges/gaps across the result, discussion and conclusion sections. Respondents within marital union shouldn’t necessary be categorized as “risky sexual practice” if they aren’t using condom within the union unless they are sero-discordant. However, the operational definition doesn’t qualify so. This resulted in a very higher proportion of respondents as a risky sexual practices (80%) which is not comparable with some local studies done in country (39% and 38% in Addis Ababa and Gondar respectively……line 205-206). This issue affects finding in the result (including the regression model), discussion, and ultimately the conclusion. Table-1 (Line 143) demonstrates the importance of this issue as 65% of the respondents are married.
- Line 157-158= It would have been good to use Viral Load levels instead of CD4 since the latter has been phased out as a treatment outcome monitoring platform for PLHIV on treatment unless this data collection was done before the change of protocol.
- Line 163= Says “four out of five respondent” which is 80% of respondents engaged in risky sexual practice, is this to say at least one of the operationally defined risky practices? If so it has to be clearly articulated so across the manuscript.
- Line 164-168= All risky sexual practices are less than 27% except consistent condom use (77.8%) which has exaggerated the overall finding as mentioned above.
- Line 169=58.8% respondents’ partners’ are sero-positive, this statement is misleading unless the term “partner” is defined well and the denominator is mentioned. As it stands now, it mean more than 40% of PLHIV on treatment are in a sero-discordant relationship which is unrealistically high. The same clarity is required for partner notification on the same line (169).
- Line 186= Table-3 Bivariate and Multivariate LR analysis, has core deficiency and need to be remodeled once the operational definition issue is addressed.
- Line 190-193= The remarks based on the finding is unrealistic
- Line 193-195= There is inconsistency between the finding and subsequent remark
- Line 201= see comment (feedback) on Line 163
- Line 205-206= The discussion pointed out important issue about the exaggerated risk level by comparing it with other studies (Addis Ababa and Gondar) and one of the authors’ possible justification is the way risk sexual practice is defined which I presume is true. This purports the need to re-consider the operational definitions of risky behavior/practices based on evidence and standard definitions.
- Line 207-211= The discussion and final remark is not clear
- Line 212-219= The discussion lack clarity and coherence. The final remark is unrelated to issues mentioned in the preceding discussion and very stretched above and beyond the scope of the study.
