# Supplementary material for: High level risky sexual behavior among persons living with HIV in the urban setting of the highest HIV prevalent areas in Ethiopia: Implications for interventions
Source: PLoS One. 2020 Nov 25;15(11):e0242701. doi: 10.1371/journal.pone.0242701 (PMC7688102; doi:10.1371/journal.pone.0242701)
Supplement: Attachment — Submitted filename: Parecer_eng.docx [file pone.0242701.s004.docx]

I do **not** authorize the disclosure of my name. I want the revision to remain anonymous. My suggestion is to accept the manuscript, but modifications are required.

The paper “High level of risky sexual behavior among persons living with HIV in the urban setting of the highest HIV prevalence in Ethiopia: Implications for HIV interventions” is very interesting. It is important to note that the authors were careful in writing and that the text has great potential for publication, in addition to dealing with a relevant theme in the literature. Some comments, however, are necessary to improve the quality of the manuscript.

The background section outlines the research problem and talks about the issue of HIV and the problem in Africa, but three essential are missing: **i)** What hypothesis do the authors intend to test? **ii)** How does the article contribute to the literature? **iii)** How is the article innovative? I suggest the authors include those answers in the final background so that the readers will better understand the purpose of the manuscript.

In line 120, the authors talk about the inclusion of variables with p <0.25. What is the reason for this value? The authors do not explain this. Are there any texts in the literature that use this value? If so, please include them in the manuscript.

In the results section, the authors should better explain Table 1 and the implication of the relative frequency on the sample on the results. As for income, I suggest adding a note explaining the values that represent poverty and extreme poverty.

In line 163, the authors write “About four fifth of the respondents had engaged in risky sexual practices”, but I did not find this information in the tables. I suggest that the authors include a table with risky sexual behaviors and the proportion of the sample with each behavior. I believe the table is more important than the text for the readers to understand the actual problem. Authors can explore Figure 1 further as well.

In Table 3, I suggest compiling the information about relationships in “lives with a partner or not”. You see, it is just a suggestion to turn the variables into just a binary. It is up to the authors to accept the suggestion or not. Line 187, table note, has only one asterisk, for p <0.05. Authors should separate p <0.05 and p <0.01, as they represent different levels of significance.

The discussion is the best part of the article, the authors manage to associate their findings with the literature. The conclusions properly finish the article.
